# Hematological, coprological and tracheoscopy results in pheasants (*Phasianus colchicus*) experimentally infected with *Syngamus trachea*

**DOI:** 10.2478/helm-2025-0019

**Published:** 2025-11-26

**Authors:** V. Vrabec, A. Königová, Z. Vasilková, E. Sesztáková, F. Humeník, P. Lazár, L. Molnár

**Affiliations:** 1 Clinic of Birds, Exotic and Free Living Animals, University of Veterinary Medicine and Pharmacy in Košice, Komenského 73, 041 81 Košice, Slovakia; 2 Institute of Parasitology, Slovak Academy of Sciences, Hlinkova 3, 040 01 Košice, Slovakia; 3 Department of Morphological Disciplines, University of Veterinary Medicine and Pharmacy in Košice, Komenského 73, 041 81 Košice, Slovakia; 4 Department of Breeding and Diseases of Game, Fish and Bees, Ecology and Cynology, University of Veterinary Medicine and Pharmacy in Košice, Komenského 73, 041 81 Košice, Slovakia

**Keywords:** pheasant, *Syngamus trachea*, hematology parameters, endoscopy

## Abstract

This study was conducted to determine hematological changes in two different age groups of pheasants (*Phasianus colchicus*) experimentally infected with 3 and 5 earthworms, 200 embryonated eggs of *Syngamus trachea*, and control groups. Comparing the hematological parameters, EPG values, and tracheoscopy findings revealed differences related to the age of the experimental birds. The most significant changes in RBC, Hb, and eosinophils (p <0,05) were found in a group of young pheasants fed with five earthworms, followed by three earthworms, and finally with 200 embryonated *S. trachea* eggs. In a group of adult pheasants, a decline in RBC and Hb was observed in groups fed 3 or 5 earthworms. The group fed with 200 embryonated eggs showed no significant difference. The hematological results revealed that the mean values of Hb and RBC were higher (P ≤ 0.05) in noninfected birds compared to infected ones. Comparison of the EPG values related to tracheoscopic findings confirmed a higher parasitic burden as well as a higher number of adults. The highest EPG value observed (1500) corresponded with the number of adults of *S. trachea* (4 pairs) recorded in a group of young pheasants fed five earthworms, with an average of 2.1 adult pairs in the trachea and an average EPG value of 750. Additionally, it was confirmed that young birds are more susceptible to S. trachea infection than adults. Based on the results, it was concluded that some hematological values were influenced by age. The differences between the pheasants and the data obtained in this study could help establish baseline values for hematological parameters in pheasants regarding the parasitic burden caused by *S. trachea*.

## Introduction

Syngamosis is a severe, pathogenic parasitic infection caused by the nematode *Syngamus trachea* (Ruff, 1999) and belongs to the Family Syngamidae and the Superfamily Strongylidae. It has been recovered from many gallinaceous and passerine species (Morgan & Clapham, 1934; Campbell, 1935; Goble & Kutz, 1945; Bandelj *et al*., 2015) with successful experimental transfer between species (Clapham, 1938). Among the parasites that affect pheasants, *Syngamus trachea* (Montagu, 1811), as cited by Chapin (1925), is one of the most pathogenic and economically significant, which results in high morbidity and mortality. In particular, it can cause significant production losses, poor weight gain, and even mortality in heavily infected birds (Ruff, 1999; Krone *et al*., 2007; Atkinson *et al*., 2008). However, very little is known about the parasite’s biological dynamics, transmission, and direct health effects on pheasants (*Phasianus colchicus*). Particularly, there is a lack of information about the dynamic changes and hematological profile of pheasant blood during parasitic infestation with *S. trachea*.

Syngamosis is most frequently observed in breeding and rearing establishments that use outdoor pens for breeding pheasants (Akand *et al*., 2020). The life cycle of the gapeworm *S. trachea* is peculiar in that transmission from bird to bird may be accomplished either directly (by ingesting embryonated eggs with third-stage larvae (L3)) or infective larvae L3 or indirectly (by ingestion of earthworms containing free or encysted gapeworm larvae they had obtained by feeding on contaminated soil). Artificially reared game birds, especially pheasants, predominantly suffer from gape worms (Fernando *et al*., 1971), and parasite pressure, influenced by various factors encountered, creates an annual challenge for pheasant keepers to rear newly hatched pheasants in their rearing aviaries successfully. Young pheasants, despite having a full-balanced feed mixture presented, are highly omnivorous and look for invertebrates and insects as a source of natural protein, as they do in their natural habitats. The release of several-week-old (6 – 8 weeks) young, immunologically naive pheasants from indoor aviaries into outdoor pens is associated with massive picking of earthworms with encysted *Syngamus trachea* L3 larvae in their bodies from the pen soil contaminated with fecal eggs shed by the previous year’s reared pheasants. Breeding aviary soil is constantly contaminated by earthworms, which become prey for young pheasants that feed on invertebrates and insects. Earthworms serve as transport (paratenic) hosts. Larvae have been shown to remain viable for more than 3 years encapsulated in earthworm muscles. However, in natural conditions, it is unclear as to what proportion of earthworms within a release pen are indeed infected, and even what proportion of those infected earthworms are ingested by pheasants (Gethings, 2018). The fact that the eggs hatch inside the earthworms and that the hatched larvae migrate through the intestinal mucosa and encyst in the muscle tissue is noteworthy. If the earthworm were not an important factor, then there would be no further hatching inside a non-avian host. It is possible that, either in the past or in the future, natural selection may favor earthworms as the primary method of transmission. (Gethings, 2018). As a result, the modes of *Syngamus trachea* eggs can affect the manifestation of clinical symptoms. There is marked pathology associated with infection by *S. trachea* (Fernando *et al*., 1971; Nevarez *et al*., 2002; Atkinson *et al*., 2008), and mortality rates of affected birds can be as high as 80 % (Wójcik *et al*., 1999). It is estimated that the average mortality rate of birds infected with *S. trachea* is approximately 25 % (Andreopoulou *et al*., 2012). The developmental stages of *S. trachea* damage the integrity of the intestinal wall, liver, and trachea, which can cause local inflammatory responses that affect hematological parameters in infected birds.

Despite the large number of publications that study hematological and biochemical parameters in poultry, there is limited knowledge about these parameters in feathered game, including the common pheasant. Moreover, the role of paratenic hosts is often underestimated in many studies, and direct methods of infecting pheasants are preferred.

The primary purpose of this study was to investigate the changes in values of hematological parameters in comparison with the intensity of S. trachea infection in two different age groups of ringnecked pheasants experimentally infected, utilizing two different methods: either with inoculated S. trachea eggs or a third-stage S. *trachea* larvae encysted in the earthworm’s body.

This paper also aimed to highlight the significant role of paratenic hosts in the transmission of syngamosis in naive pheasants in rearing pens, where regular rotations are not possible.

## Materials and Methods

### Geographical area

The pheasants used in the experiments originated from the pheasantry farm “Samostatná bažantnica in Rozhanovce”, 48°46’18”N, 21°22’21”E, owned by the University of Veterinary Medicine and Pharmacy in Košice, Slovak Republic. It involves intensive breeding of pheasants in aviaries, with an annual production of approximately 6,000 pheasants.

### The Birds and Their Treatment

Experiment I was conducted from March to May 2022 on 10-month-old, sexually mature common ring-necked pheasants that were hatched and reared the previous year with the intention of reproduction. Forty pheasants (divided into four groups – A, B, C, D; each with 10 individuals and a sex ratio of 1 male to 9 females) were housed separately in breeding aviaries. All the birds were tagged with identification numbers, placed on the wing web, and dewormed with flubendazole (Flubenvet), administered in a feed mixture at a dose of 60 ppm over 7 days. One week after deworming, all the birds were short-term anesthetized with isoflurane, examined endoscopically (tracheoscopy) (Storz, 2.7mm rigid endoscope) to exclude the presence of adult *Syngamus trachea* nematodes in their trachea, blood samples were collected into a 1cc Li-heparin container by *jugular vein* puncture, and fecal samples were obtained by *exploration per cloacam*, respectively. Both procedures were performed according to long-established and proven procedures (Westerhof, 1995; Chan *et al*., 2013).

Experiment II was conducted from June to August 2022 on young, immunologically naive 8-week-old pheasants who were relocated from indoor aviaries to outdoor release pens. The design of the study was identical to that used in adults.

The legends of Tables 1 and 2 provide a detailed description of the experimental groups.

**Table 1.** Hematological findings in adult pheasants.

ADULT PHEASANTS (11 ms)	Ec (T/l) Mean ± SD	Ht (1/1)	Hb (g/l)	Le (G/l)	He (%)	Eo (%)	Ba (%)	Ly (%)	Mo (%)
**A 1**	2.64 ± 0.234	0.396 ± 0.013	103.7 ± 6.914	29.5 ± 4.227	34.1 ± 6.58	0.60 ± 0.49	0.6 ± 0.49	64.1 ± 6.17	0.6 ± 0.49
**A**	2.525 ± 0.194	0.384 ± 0.013	100.5 ± 5.937	31.2 ± 3.627	33.8 ± 9.64	1.40 ± 0.66	0.7 ± 0.64	63.3 ± 9.34	0.8 ± 0.63
t-test									
Paired t test									
P value	p<0.0001	p<0.0001	0.0003	0.0020	0.8056	0.0031	0.7142	0.5395	0.4486
P value summary	***	***	***	**	ns	**	ns	ns	ns
Are means signif.different? (P<0.05)	Yes	Yes	Yes	Yes	No	Yes	No	No	No
**B 1**	2.471 ± 0.206	0.397 ± 0.015	103.9 ± 6.204	28.0 ± 2.863	35.6 ± 8.59	0.6 ± 0.66	0.8 ± 0.75	61.2 ± 8.68	0.4 ± 0.49
**B**	2.27 ± 0.159	0.396 ± 0.012	93.4 ± 3.072	31.0 ± 3.065	35.6 ± 8.52	1.6 ± 0.92	0.8 ± 0.75	61.6 ± 8.31	0.4 ± 0.49
t-test									
Paired t test									
P value	0.0026	0.7577	p<0.0001	0.0067	1.0000	0.0038	1.0000	0.1039	1.0000
P value summary	**	ns	***	**	ns	**	ns	ns	ns
Are means signif.different? (P<0.05)	Yes	No	Yes	Yes	No	Yes	No	No	No
**C 1**	2.954 ± 0.3	0.395 ± 0.017	104.8 ± 7.871	28.3 ± 4.605	28.5 ± 5.82	0.5 ± 0.5	0.8 ± 0.75	69.2 ± 6.16	1 ± 0.77
**C**	2.877 ± 0.283	0.397 ± 0.012	106.7 ± 7.10	30.6 ± 3.104	31.4 ± 3.58	0.8 ± 0.75	0.6 ± 0.49	64.4 ± 5.68	0.8 ± 0.75
t-test									
Paired t test									
P value	0.1226	0.7435	0.0973	0.0531	0.2191	0.2789	0.5109	0.0056	0.5843
P value summary	ns	ns	ns	ns	ns	ns	ns	**	ns
Are means signif.different? (P<0.05)	No	No	No	No	No	No	No	Yes	No
**D 1**	2.833 ± 0.195	0.396 ± 0.017	102.7 ± 5.254	32.1 ± 2.30	33.1 ± 3.24	0.6 ± 0.66	0.4 ± 0.49	65.1 ± 3.21	0.8 ± 0.6
**D**	2.814 ± 0.219	0.386 ± 0.016	102.1 ± 4.369	32.5 ± 2.418	32.2 ± 2.23	0.6 ± 0.66	0.4 ± 0.49	66.2 ± 2.36	0.6 ± 0.66
t-test									
Paired t test									
P value	0.7115	0.1679	0.6748	0.6959	0.4036	1.0000	1.0000	0.2919	0.5109
P value summary	ns	ns	ns	ns	ns	ns	ns	ns	ns
Are means signif.different? (P<0.05)	No	No	No	No	No	No	No	No	No

**TTable 2.** Hematological findings in young pheasants.

YOUNG PHEASANTS (8 ws)	Ec (T/l) Mean ± SD	Ht (1/1)	Hb (g/l)	Le (G/l)	He (%)	Eo (%)	Ba (%)	Ly (%)	Mo (%)
**A 1**	2.88 ± 0.145	0.389 ± 0.016	101.6 ± 4.20	32.4 ± 3.168	29.9 ± 4.48	0.8 ± 0.6	0.6 ± 0.49	68.2 ± 3.74	0.5 ± 0.5
**A**	2.745 ± 0.14	0.381 ± 0.012	98.4 ± 3.072	33.9 ± 2.467	28.3 ± 3.29	1.5 ± 0.67	0.7 ± 0.46	68.8 ± 3.09	0.7 ± 0.46
t-test									
Paired t test									
P value	p<0.0001	0.0031	0.0004	0.0384	0.0330	0.0445	0.6601	0.3133	0.388
P value summary	***	**	***	*	*	*	ns	ns	ns
Are means signif.different? (P<0.05)	Yes	Yes	Yes	Yes	Yes	Yes	No	No	No
**B 1**	2.903 ± 0.211	0.394 ± 0.013	101.6 ± 4.521	32.5 ± 3.354	31.5 ± 3.07	0.6 ± 0.663	0.6 ± 0.49	66.4 ± 3.23	0.9 ± 0.94
**B**	2.645 ± 0.186	0.387 ± 0.014	95.2 ± 2.675	33.1 ± 3.30	33.6 ± 3.26	1.6 ± 0.916	0.8 ± 0.6	63.2 ± 4.38	0.9 ± 0.7
t-test									
Paired t test									
P value	p<0.0001	0.2848	0.0005	0.4679	0.0029	0.0229	0.4486	0.0054	1.0000
P value summary	***	ns	***	ns	**	*	ns	**	ns
Are means signif.different? (P<0.05)	Yes	No	Yes	No	Yes	Yes	No	Yes	No
**C 1**	2.94 ± 0.213	0.386 ± 0.019	103.3 ± 7.563	33.0 ± 3.464	31.3 ± 4.34	0.5 ± 0.5	0.6 ± 0.49	67.0 ± 4.47	0.6 ± 0.49
**C**	2.602 ± 0.174	0.391 ± 0.014	96.6 ± 4.841	34.2 ± 1.72	33.1 ± 4.18	1.2 ± 0.6	0.5 ± 0.5	64.6 ± 4.50	0.6 ± 0.49
t-test									
Paired t test									
P value	p<0.0001	0.4525	0.0003	0.1475	0.0744	0.0013	0.6733	0.0351	1.0000
P value summary	***	ns	***	ns	ns	**	ns	*	ns
Are means signif.different? (P<0.05)	Yes	No	Yes	No	No	Yes	No	Yes	No
**D 1**	2.8 ± 0.201	0.399 ± 0.014	100.4 ± 7.618	31.3 ± 2.90	30.8 ± 3.46	0.6 ± 0.489	0.7 ± 0.64	67.2 ± 3.43	0.7 ± 0.46
**D**	2.86 ± 0.241	0.405 ±0.01	100.0 ± 5.059	30.9 ± 2.165	32.5 ± 4.15	0.7 ± 0.64	0.6 ± 0.49	65.7 ± 3.82	0.5 ± 0.5
t-test									
Paired t test									
P value	0.0299	0.3288	0.7239	0.5744	0.2745	0.6783	0.7142	0.2932	0.388
P value summary	*	ns	ns	ns	ns	ns	ns	ns	ns
Are means signif.different? (P<0.05)	Yes	No	No	No	No	No	No	No	No

### Hematological examination

One ml of blood was collected from the jugular vein into a Lithium-Heparin tube. A blood smear was prepared from a blood droplet without heparin. The values of total red blood cells (RBC), total white blood cells (WBC), packed cell volume (PCV), hemoglobin content (Hb), and the differential count of white blood cells of blood smears were determined. Based on RBC, PCV and Hb, values of MCV (mean corpuscular volume), MCH (mean corpuscular hemoglobin) and MCHC (mean corpuscular hemoglobin concentration) were calculated (Campbell,1995). The numbers of red and white blood cells were counted in Bürker’s hemocytometer, after mixing with Natt-Herrick solution in a 1:200 ratio (Natt & Herrick, 1952). Hemoglobin content was determined by spectrophotometry (at 540 nm) after the blood was mixed with Drabkin solution in a 1:250 ratio (Drabkin, 1945). Blood smears were stained with Hemacolor (Miles Laboratories, Inc., Elkhart, Indiana, USA). One hundred white blood cells were evaluated for each smear. The type of blood cells was determined according to the method described by Lucas and Jamroz (1961). For each variable of interest, we present the mean value and standard deviation (SD) in classification by gender and age (P > 0.005).

For the statistical analysis, all variables (excluding percentage values) were analyzed using the paired t-test and one-way ANOVA combined with Tukey’s multiple comparison test.

### Paratenic hosts

Two species of earthworms, *Eisenia foetida* and *Lumbricus terrestris*, were collected from the soil of pens used annually for pheasant rearing. Due to limited spatial capacities, aviary rotation between individual breeding seasons is not possible; therefore, the persistence of paratenic hosts in the aviary soil is almost 100 %.

### Keeping the infested earthworms under laboratory conditions

The earthworms harvested, in numbers ranging from tens to hundreds, were maintained under laboratory conditions in plastic containers with a volume of 50 liters, filled with the soil directly taken from the release pens. The soil was kept moist. The earthworms were transferred to fresh soil after a week. It was estimated that all the soil would be discarded, along with any free eggs or worm larvae present in the earthworms’ guts, and thus incubated. The earthworms in the soil were given feces positive for *Syngamus trachea* eggs taken from the aviaries and confirmed positive by the flotation method examination. The feces were placed on the surface of the soil, and earthworms ate them by regularly plowing the soil.

### The earthworms examinations

The earthworms were examined for the presence of eggs and larvae of *Syngamus trachea*: parasitological dissection of the earthworm body with the capture of the contents of the entire digestive tract, similarly to the fecal examination, the contents of the digestive tract were examined by coprological flotation methods and qualitative and quantitative propagation stages of *Syngamus trachea* were identified during examination of the contents of the digestive tract of earthworms (Clapham, 1997). The body walls of earthworms were examined through a thorough microscopic evaluation of compression slides to confirm the positive findings of encysted L3 larvae in hyaline cysts within the earthworms’ bodies.

### Tracheal Endoscopy

Short-term inhalant anesthesia with isoflurane was applied to the birds in individual groups to examine the trachea. Tracheoscopy to the level of the syrinx was possible in medium- to large-sized birds using a 180 mm long, 2.7 mm endoscope. Visualization could be improved by extending the pheasant neck (Murray *et al*., 1999). Simultaneously, the blood was withdrawn from the jugular vein.

### Parasitological methods

The quantitative coprological McMaster method, with a minimum detection limit of 50 EPG (eggs per gram), using Sheather’s flotation solution (density 1.280), was used to detect the presence of *S. trachea* eggs (Coles *et al*., 1992).

### Parasitological necropsy

**Fig. 1.**
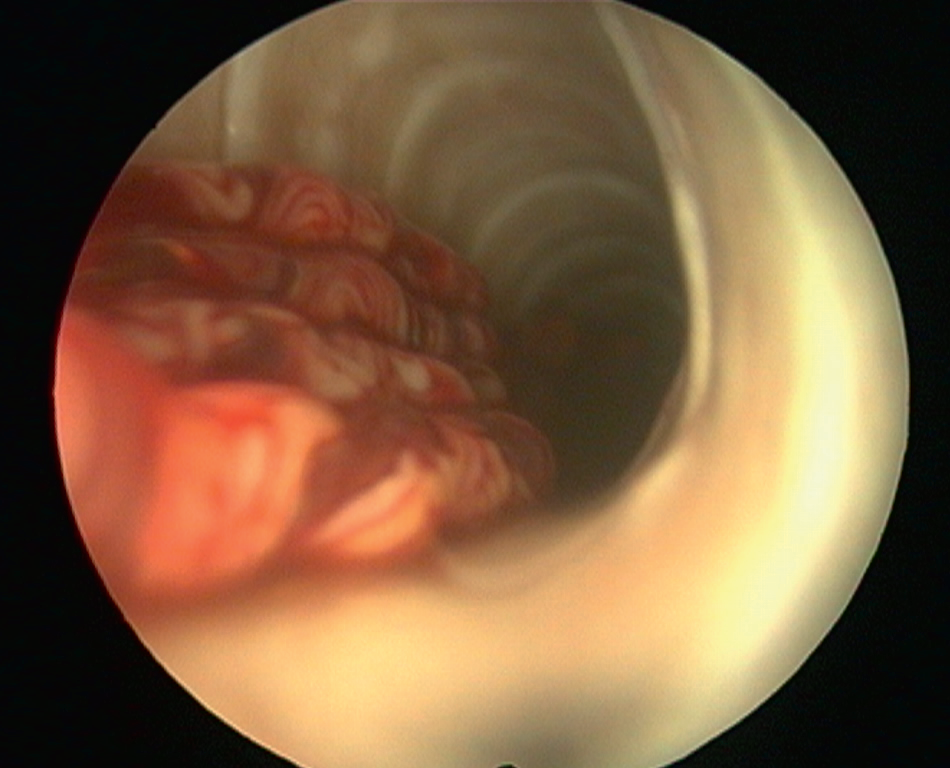
Tracheoscopic finding of *Syngamus trachea* adults fixed to tracheal mucosal lining.

**Fig. 2.**
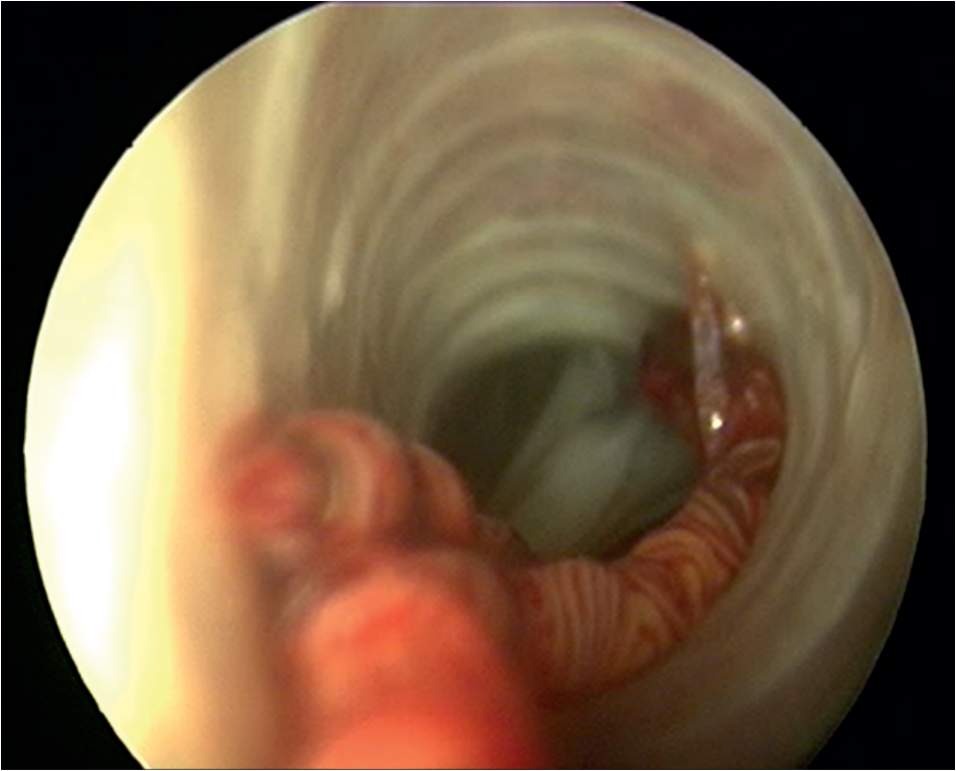
Adult gapeworms fi xed in lower part of trachea, bifurcation seen.

**Fig. 3.**
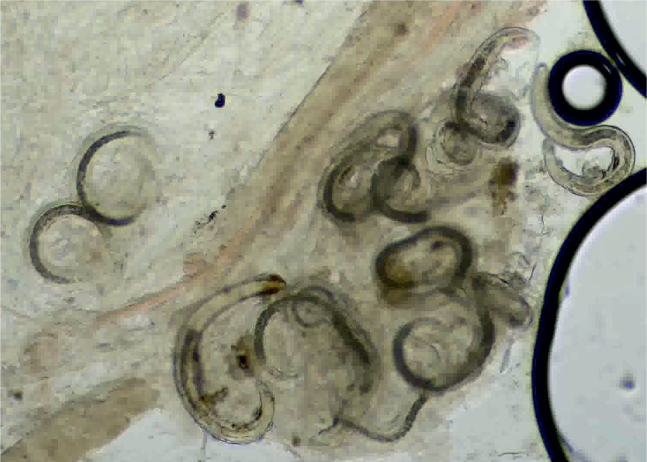
Infectious L3 larvae of *Syngamus trachea* encysted in the body wall of earthworm – compression microscope slide, magnification 10x.

A parasitological necropsy of a bird involves a detailed post-mortem examination to identify internal and external parasites. In short, the process begins with external examination for lesions or discharges, followed by incisions to the body cavity to assess internal organs, air sacs, and the gastrointestinal tract for parasites, abnormalities, and changes in tissue consistency, size, or color. Samples of organs and contents are collected for further analysis, including microscopic examination for parasites (Van Riper, 1980). Figures 1 – 2 show the trachea *in situ*, highlighting the presence of *S. trachea* within its structure. Figure 3 illustrates infectious larvae L3 encysted in the earthworm’s body wall.

## Ethical Approval and/or Informed Consent

The Ethics Committee of the Institute of Parasitology, Slovak Academy of Sciences, approved the use of animals for this study. All procedures performed in this study were conducted in accordance with the ethical standards of the Ethics Committee, as outlined in the Animal Welfare Act No. 23/2009, and met the requirements of the Ethics Committee of the Institute of Parasitology of the Slovak Academy of Sciences, as established on 17 November 2021. The study was approved on 1 January 2022.

## Results

### Endoscopy and parasitology results

**Fig. 4.**
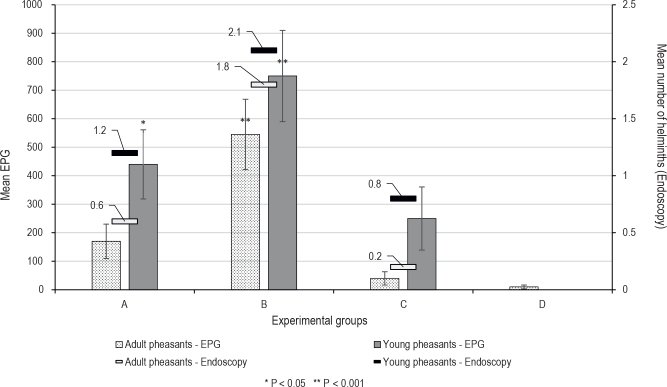
Summary of endoscopic and EPG examinations in young and old pheasants. A - group infected with three earthworms; B - group infected with five earthworms; C - group infected with 200 embryonated eggs; D - control group

The results obtained from the tracheoscopic examinations and mean EPG findings of both young and adult pheasants are presented in Figure 4. In two young pheasants from the group B, the tracheoscopic findings were negative, and in two pheasants, the findings included four pairs of adults in the trachea. Three adults were detected in two pheasants, two pairs of adults were found in three pheasants, and one pair of adults in one pheasant. The maximum EPG value of 1500 was found in one pheasant, which also had four adults in the trachea. EPG 1200 was found in two pheasants, EPG 1000 in one, EPG 800 in two, EPG 600 and 400 in one pheasant, and EPG 0 was found in two pheasants.

Findings in groups infected with three earthworms showed average EPG values of 440 in the “young pheasants” group, compared to 170 in the “adult pheasants” group. Endoscopic findings were an average of 1.2 adults per pheasant in the young group versus 0.6 adults in the adult pheasants group. In group C, inoculated with 200 embryonated eggs of *S. trachea*, an average of 0.8 adults were found in the group of young pheasants, compared to 0.2 adults in the group of adult pheasants. The average EPG values were 250 in the group of young pheasants compared to an EPG of 40 in the group of adult pheasants. In the group of young pheasants, the trachea was endoscopically negative in 6 individuals. One pheasant had one adult, two pheasants had two adults, and one pheasant had three adults. In adult pheasants from group C, the endoscopic findings were negative in 8 individuals, and one adult was found in two pheasants.

### Hematology

Hematological findings in groups of adult pheasants are shown in Table 1 in individual groups Aa1 (adult pheasants before inoculation of 3 infectious earthworms), group Aa (adult pheasants after inoculation of 3 infectious earthworms), with values Mean ± SD for Ec 2.64±0.234 T/l (A1) vs Ec 2.525 ±0.194 T/l (A).

In group Ba1 (adult pheasants before inoculation of 5 infectious earthworms), RBC values were 2.471 ± 0.206 vs Ba (adult pheasants after inoculation of 5 infectious earthworms), 2.27 ± 0.159 T/l. In group Ca1 (adult pheasants before administration of 200 eggs of *Syngamus trachea*), RBC was 2.954 ± 0.3 vs. Ca (adult pheasants after administration of 200 eggs), 2.877 ± 0.283 T/l. In group Da1, Da (adult pheasants – controls), the following numbers of RBC were 2.833 ± 0.195 T/l vs. D1, 2.814 ± 0.219 T/l.

Hematocrit values in individual groups of adult pheasants are shown as follows: Aa1 0.396±0.013 l/l vs Aa 0.384 ± 0.013, group Ba1 0.397 ± 0.015 l/l vs Ba 0.396 ±0.012 l/l, group Ca1 0.395 ± 0.017 vs Ca 0.397± 0.012 l/l and group Da1 0.396 ± 0.017 vs Da 0.386 ± 0.016 l/l.

Amounts of hemoglobin expressed as Mean ± SD (g/l) according to individual groups were as follows: Aa1 103.7 ± 6.914 vs. Aa 100.5 ± 5.937, group Ba1 103.9 ± 6.204 vs Ba 93.4 ± 3.072, group Ca1 104.8 ± 7.871 vs Ca 106.7 ± 7.1 and group Da1 102.7 ± 5.254 vs Da 102.1 ± 4.369.

The number of WBC was slightly increased in individual groups Aa1 29.5 ± 4.272 vs. Aa 31.2 ± 3.627 G/l (p< 0.05), in group Ba1 28 ± 2.863 vs. Ba 31 ± 3.065 G/l (p< 0.05), group Ca1 28.3 ± 4.605 vs Ca 30.6 ± 3.104 G/l (ns) and in group Da1 32.1 ± 2.3 vs Da 32.5 ± 2.418 G/l (ns).

The values of the differential blood count (He, Ba, Ly, Mo) are shown in the Table 1 as Mean ± SD in percentage (Table 1).

The RBC values in % were as follows in the monitored groups: Aa1 0.60 ± 0.489 vs. Aa 1.40 ± 0.663 (p<0.05), in group Ba1 0.6 ± 0.663 vs. Ba 1.60 ± 0.916 (p<0.05), in in group Ca1 0.5 ± 0.5 vs Ca 0.8 ± 0.748 (ns) and group Da1 0.6 ± 0.663 vs Da 0.6 ± 0.663 (ns).

### Young pheasants

Infection induced by inoculation of 3 and 5 earthworms and inoculation of 200 *Syngamus trachea* eggs caused a significant decrease in RBC in groups of young pheasants (all groups p<0.001) Hematological findings in groups of young pheasants are shown in Table 2. in individual groups Ay1 (young pheasants before inoculation of 3 infectious earthworms) vs group Ay (young pheasants after inoculation), with mean ± SD values for RBC 2.88 ± 0.145 T/l (Ay1) vs RBC 2.745 ± 0.14 T/l (Ay). In group By1 (before inoculation of 5 infectious earthworms), RBC values were 2.903 ± 0.211 vs By (after inoculation of 5 infectious earthworms) 2.645 ± 0.186 T/l.

In group Cy1 (before administration of 200 eggs of *Syngamus trachea*), RBC was 2.94 ± 0.213 vs Cy (after administration of 200 eggs) 2.602 ± 0.174 T/l. In group Dy1, Dy (control), the following numbers were obtained: Ec 2.8 ± 0.201 T/l vs Dy 2.86 ± 0.241 T/l. Hematocrit values in individual groups of young pheasants are: Ay1 0.389 ± 0.016 vs. Ay 0.381 ± 0.012 l/l (p<0.05), group By1 0.394 ± 0.013 l/l vs By 0.387 ± 0.014 l/l (ns), group Cy1 0.386 ± 0.019 vs Cy 0.391 ± 0.014 l/l (ns), and group Dy1 0.399 ± 0.014 vs Dy 0.405 ± 0.01 l/l (ns).

Amounts of hemoglobin expressed as Mean ± SD (g/l) according to individual groups were: Ay1 101.6 ± 4.2 vs. Ay 98.4 ± 3.072 (p<0.05), group By1 101.6 ± 4.521 vs By 95.2 ± 2.675 g/l (p<0.05), group Cy1 103.3 ± 7.563 vs Cy 96.6 ± 4.841(p<0.05), and group Dy1 100.4 ± 7.618 vs Dy 100 ± 5.059 g/l (ns).

The number of WBC was slightly increased in groups Ay1 32.4 ± 3.168 vs. Ay 33.9 ± 2.467 G/l (p<0.05), in group By1 32.5 ± 3.354 vs By 33.1 ± 3.3 G/l (ns), group Cy1 33 ± 3.464 vs Cy 34.2 ± 1.72 G/l (ns) and in group Dy1 31, 3 ± 2.9 vs. Dy 30.9 ± 2.165 G/l (ns). The values of the differential blood count (He, Ba, Ly, Mo) in young pheasants are shown in Table 2, with the mean and standard deviation (SD) in percentage. See the table. Heterophils in group Ay1 were 29.9 ± 4.48 % vs Ay 28.3 ± 3.29 (p<0.05), By1 31.5 ± 3.07 vs By 33.6 ± 3.26 (p<0.05), group Cy1 31.3 ± 4.34 vs Cy 33.1 ± 4,18 (ns) and group Dy1 30.8 ± 3,46 vs Dy 32.5 ± 4.15 (ns).

The eosinophils (Eo in %) in the monitored groups of young pheasants were: Ay1 0.8 ± 0.6 vs. Ay 1.5 ± 0.67 (p<0.05), in group By1 0.6 ± 0.663 vs By 1.6 ± 0.916 (p<0.05), in group Cy1 0.5 ± 0.5 vs. Cy 1.2 ± 0.6 (p<0.05) and group Dy1 0.6 ± 0.489 vs Dy 0.7 ± 0.64 (ns).

## Discussion

Syngamosis, a disease caused by the parasite *Syngamus trachea*, poses a significant health issue in poultry farms and wild birds, particularly in areas where the density of susceptible animals is high. The present work presents the impacts of experimental infection in adult (10 months old) and young pheasants (8 weeks old) with 3 and 5 infected earthworms and 5 mL of inoculum containing 200 embryonated eggs of *S. trachea*, as evaluated through complete hematological, coprological, and tracheoscopic examinations.

When monitoring and assessing hematological indicators, it is essential to consider the disease’s pathogenesis. After the consumption of embryonated infectious eggs or a paratenic host, the infectious L3 larva is released into the intestine of the definitive host, which subsequently migrates through the intestinal mucosa into the blood and is carried to the lungs and then to its definitive site - the trachea (Winward & Russell, 1976). Already, during the penetration of the intestinal wall and the passage of the larva through the bloodstream, an inflammatory reaction of the infested organism can occur, which is also reflected in the hematological status by an increase in the number of leukocytes (Gethings *et al*., 2015a,b) what was also observed in our study, where all three experimental groups showed an increase in the number of white blood cells, primarily eosinophils. Eosinophilia in the experimental groups increased by 90 – 150 % compared to the control group. Eosinophilia has been described as one of the fundamental changes in the hematological picture during parasitic infections in vertebrates. The principle and mechanism of this phenomenon can be described from several perspectives (Rosenberg *et al*., 2013). In parasitic infections of animals, a strong Th2-induced immune response occurs, which is accompanied by metabolic and physical interactions between the host and the worm. The effector mechanisms that eliminate the worms from the body vary depending on the habitat and niche of the parasite. Best understood in the intestine, in some cases, goblet cells are key; in others, mast cells or antibodies play an essential role in host defense. Immune mediators can cause crowding, deprive the worm of energy or nutrients, or disrupt its habitat (Huang & Appleton, 2016). In particular, IL-5 plays a central role in inducing eosinophil activation and recruitment to sites of infection, while eotaxin also promotes eosinophilia (Pelaia *et al*., 2019). This phenomenon is also supported by earlier studies that demonstrated the ability of eosinophils to kill nematode larvae in vitro (Capron *et al*., 1979). Changes in the hematological picture during helminth infection are also notable, particularly in the red blood cell count, as evidenced by the values of erythrocytes, hemoglobin, and hematocrit (Varga, 1971). The same was also observed in our study. However, changes in the blood count concerning the red blood cell count are not as striking as those in the case of eosinophils, which are related to the organism’s immune response. The very principle of changes concerning the hematocrit, the number of erythrocytes and hemoglobin values is primarily due to the parasitic *Syngamus trachea* way of life, when blood-sucking by the parasite occurs and also the disruption of the integrity of the tracheal wall and the associated formation of hemorrhagic lesions (Meister *et al*., 2022). When assessing hematological indicators, it is essential to note that changes in the hemogram in birds and poultry occur even under physiological conditions throughout the year and are related to the season, training, and nutrition. It is also necessary to note the differences between red and white blood cells, which vary depending on the individual’s age. In young individuals, we observe lower values of PCV, Hb, and RBC than in adult individuals of the same species. However, the numbers of polychromatophils and normoblasts were higher in young animals (Hrabčáková *et al*., 2014).

The pathology of *Syngamus trachea* also consists of the obstruction of the respiratory tract by adults (Martin *et al*., 2020). In our study, we determined the number of adults by endoscopic examination. The primary clinical significance of McMaster EPG is its ability to provide a numerical estimate of the number of parasite eggs in a given amount of feces. This allows for a more objective assessment of infection intensity than a simple qualitative fecal flotation test (Bondarenko *et al*., 2009). Utilizing quantitative methods such as McMaster, it is possible to assess the accuracy and effectiveness of anthelmintic treatment, which is also closely related to the potential for anthelmintic resistance (Tsukahara *et al*., 2017). On the other hand, in livestock, regular EPG testing helps manage parasite control programs, allowing producers to identify high-shedding individuals or groups of animals and implement targeted treatments or management strategies to reduce the overall parasitic load (McEvoy *et al*., 2024). Egg production by adult worms also plays a vital role in the epidemiology of the disease. We determined the number of eggs produced by coprological examination of feces by the quantitative McMaster method, where the highest EPG values, the average for the group of 750 and the average number of adults (2.1) present in the trachea, were found in group B (young pheasants).

The EPG values in the group of young pheasants were, on average, 250, with six values being negative. In the group of adults, the endoscopy was negative in 8 individuals, and in two cases, one adult was found to have a tracheal lesion. EPG values of 0 were found in 7 individuals. Groups D (young and adult) were negative tracheoscopically for the presence of *S. trachea* adults in the trachea of the observed animals, and EPG findings were also 0, except for the adult group, where EPG values of 50 were found in two individuals. Very similar results were obtained in a study focused on the prevalence of syngamosis in European starlings. Where 1.82 adults were found in the tracheae, and the prevalence reached 42 % (Chen & Sukhdeo, 2023). In domestic poultry, they may be found in high numbers, as recent studies have reported the presence of 12 – 48 adults in the trachea of hens (Gurumyen *et al*., 2020). The spread and intensity of *S. trachea* infection in pheasant farming is also influenced by climatic conditions. In general, it can be stated that high relative humidity and environmental temperature increase the risk of infection, as they positively affect the development cycle of the parasite. On the contrary, dry periods, without precipitation and with sufficient sunlight, suppress the development of infection, as the parasite eggs produced in the feces dry out (Gethings *et al*., 2015).

Several compounds have been shown effective against *S. trachea* under experimental conditions. Flubendazole is the only licensed anthelmintic for use in poultry and game birds. There are some more compounds proven to be effective such as thiabendazole, mebendazole, fenbendazole and cambendazole. Levamisole has proven effective in game birds. Even ivermectin injections may be effective in treating resistant strains but ivermectin will not kill adult gapeworms (Akand *et al*., 2020).

The above findings confirm that several factors contribute to the epidemiology of *Syngamus trachea*, including stocking density of susceptible individuals, the presence of paratenic hosts, season, breeding hygiene, general health status, and individual immunity levels (Gethings *et al*., 2015a,b; Meister *et al*., 2022).

## Conclusion

The results of this study may be helpful as a complementary diagnostic tool in pheasant clinical evaluations. They could contribute to the early detection of gapeworm infestations in pheasant farming, even when the birds are still asymptomatic. The tracheoscopic examination of the trachea may be considered a rapid intravital diagnostic method in cases involving smaller numbers of game birds, as well as at small breeding farms. The use of hematological parameters in relation to parasite burden may also be considered.
